# CK1δ Kinase Activity Is Modulated by Chk1-Mediated Phosphorylation

**DOI:** 10.1371/journal.pone.0068803

**Published:** 2013-07-04

**Authors:** Joachim Bischof, Sven-Jannis Randoll, Nadine Süßner, Doris Henne-Bruns, Lorenzo A. Pinna, Uwe Knippschild

**Affiliations:** 1 Department of General and Visceral Surgery, Ulm University Hospital, Ulm, Germany; 2 Department of Biological Sciences and Consiglio Nazionale delle Ricerche Neuroscience Institute, University of Padova, Padova, Italy; University of Padova, Italy

## Abstract

CK1δ, a member of the casein kinase 1 family, is involved in the regulation of various cellular processes and has been associated with the pathophysiology of neurodegenerative diseases and cancer. Therefore recently, interest in generating highly specific inhibitors for personalized therapy has increased enormously. However, the efficacy of newly developed inhibitors is affected by the phosphorylation state of CK1δ. Cellular kinases phosphorylating CK1δ within its C-terminal domain have been identified but still more information regarding the role of site-specific phosphorylation in modulating the activity of CK1δ is required. Here we show that Chk1 phosphorylates rat CK1δ at serine residues 328, 331, 370, and threonine residue 397 as well as the human CK1δ transcription variants 1 and 2. CK1δ mutant proteins bearing one, two or three mutations at these identified phosphorylation sites exhibited significant differences in their kinetic properties compared to wild-type CK1δ. Additionally, CK1δ co-precipitates with Chk1 from HT1080 cell extracts and activation of cellular Chk1 resulted in a significant decrease in cellular CK1δ kinase activity. Taken together, these data point towards a possible regulatory relationship between Chk1 and CK1δ.

## Introduction

The casein kinase 1 (CK1) family of evolutionary conserved serine/threonine specific kinases is ubiquitously expressed in all eukaryotic organisms [1]. In mammals seven CK1 isoforms (α, β, γ1, γ2, γ3, δ, and ε) and their splice variants have been identified which are all highly conserved within their kinase domains but differ considerably in length and sequence of their N- and C-terminal regions [1]. CK1 has been shown to phosphorylate substrates bearing either a canonical or a non-canonical consensus sequence [2]–[6]. Recently a full catalog of additional consensus sequences targeted by CK1 has been identified [7]. The ability of CK1 isoforms to recognize so many different motifs explains the still increasing number of CK1 substrates which includes key regulatory proteins playing pivotal roles in a variety of cellular processes. Processes regulated by CK1 encompass cell cycle progression and cytokinesis [8], apoptosis [9]–[13], chromosome segregation, microtubule dynamics, kinetechore and centrosome specific functions [14]–[19], DNA repair and recombination processes [20], RNA metabolism [21]–[23], Wnt signaling [24]–[29], circadian rhythm [30]–[32] and vesicle transport processes [33]–[39].

Deregulation as well as the occurrence of mutations within the coding region of CK1 family members seem to play important roles in the development of neurological disorders like sleeping disorders, bipolar I disorder, Parkinsonism-dementia complex of Guam and Alzheimer’s disease [40]–[48]. In solid tumors, changes in CK1δ expression level and activity contribute to tumorigenesis and progression [49]–[57]. Furthermore, alterations in the degree of posttranslational modifications, especially phosphorylation, might have an impact on carcinogenesis and cancer treatment. Recently it has been reported that site-specific phosphorylation also weakens the inhibitory effect of CK1δ-specific small molecule inhibitors [58], [59].

In the cell CK1 expression and activity is tightly regulated. Processes regulating CK1 activity include stimulation of cells with insulin, viral transformation, treatment of cells with topoisomerase inhibitors or γ-irradiation, all resulting in elevated CK1 activity [50], [60]–[62]. Furthermore, alternative splicing of CK1 isoforms is involved in modulating their subcellular localization, turnover and substrate binding [63]–[65]. CK1 can also be regulated by interaction with small molecules and other proteins. Interaction with cellular proteins has been shown to influence the localization of CK1 [66]–[68] and to either enhance or inhibit its activity [21], [69]. Furthermore, intramolecular autophosphorylation down-regulates CK1 activity [70]–[72]. However, several mechanisms exist to overcome the inhibitory effect of autophosphorylation [70], [71], [73].

Differences in the activity of CK1δ in different tissues and cell lines have been suggested to be partially caused by site-specific phosphorylation by cellular kinases. One of these kinases has been identified as PKA which phosphorylates CK1δ preferentially at Ser-370 *in vitro* and *in vivo* [74]. As consequence of PKA-mediated phosphorylation, CK1δ kinase activity is reduced while in turn mutation of Ser-370 to alanine enhances CK1δ-mediated substrate phosphorylation. Inhibition of the interaction of PKA with A-kinase anchoring proteins (AKAPs) in MiaPaCa-2 cells led to significantly increased CK1δ kinase activity and overexpression of the CK1δ^S370A^ mutant enhanced the formation of an ectopic dorsal axis during *Xenopus laevis* development [74]. Whereas the phosphorylation site mainly targeted by PKA has been identified, only evidence exists that additional kinases, among them AKT, Clk2, and PKC-α, are able to phosphorylate CK1δ within its C-terminal domain [74]. Given the fact that CK1δ is also involved in regulating cell cycle progression, checkpoint kinase 1 (Chk1) could also be involved in the regulation of CK1δ kinase activity by site-specific phosphorylation. This assumption is supported by the fact that several minimal consensus sequences for Chk1 are present within the C-terminal regulatory domain of CK1δ [75]. Here we show that Chk1 is able to phosphorylate rat CK1δ at Ser-328, Ser-331, Ser-370, and Thr-397 as well as human CK1δ transcription variants (TV) 1 and 2 *in vitro*. CK1δ mutant proteins bearing one, two or three mutations at the identified phosphorylation sites exhibit altered kinetic characteristics compared to wild-type CK1δ. Furthermore we show an interaction of CK1δ and Chk1 by co-immunoprecipitation analyses and that activation of cellular Chk1 by hydroxyurea results in a significant decrease in the activity of CK1δ. Therefore our data suggest a regulatory effect of Chk1 on the activity of CK1δ.

## Materials and Methods

### Extraction of RNA and cDNA Synthesis

First, RNA was extracted from the human fibrosarcoma cell line HT1080 [76] using the peqGOLD RNAPure system (Peqlab, Germany) according to the manufacturer’s instructions. 1 µg of total RNA was then reverse transcribed using oligo(dT) primers and the AffinityScript Multiple Temperature cDNA synthesis kit (Agilent Technologies, USA). For subsequent amplification of human CK1δ TV1 and 2 fragments 1 µl of cDNA synthesis reaction was used in PCR.

### Construction of Expression Vectors

Full length CK1δ and C-terminal fragments of rat CK1δ (accession ID: L07578 and [77]) were amplified by PCR using the primers listed in table 1. Resulting PCR products were first ligated into the pSC-A cloning vector (Agilent Technologies, USA) followed by subsequent cloning into pGEX expression vectors (GE Healthcare, UK) to generate the GST-CK1δ fusion proteins shown in table 2. The C-terminal fragments of human CK1δ TV1 (NM_001893) and TV2 (NM_139062) were amplified from HT1080 cDNA.

**Table 1:** Sequences of CK1δ-specific primers used for cloning and site-directed mutagenesis (SDM).

Primer	Sequence
P1	5′-CGAATTCCATGGAGCGGGAACGCCG-3′
P2	5′-GGATCCTCAGTAGGTGGTACGTCGTGG-3′
P3	5′-CCATGGGGGCCCCAGTCAACGTCTCCTCA-3′
P4	5′-CTCGAGTCAGTAGGCGGTACGTCGTGG-3′
P5	5′-AACCTGTACTTCCAGGGCGAATTCCGCGGGGCCCCC-3′
P6	5′-GAGTTAATGAATTCGCGTCATCGGTGCACGACAGACTGAAGACC-3′
P7	5′-GAGTTAATGAATTCGCGCTACTTGCCGTGGTGTTCGAAAGGAATGC-3′
SDM1	5′-CCACTCGTGGCCTCCCTGCTACAGCTTCCGGCCG-3′
SDM2	5′-GCCGGCCTTCGACATCGTCCCTCCGGTGCTCACC-3′
SDM3	5′-GGCCTCCCTGCTGCAGCTTCCGGCC-3′
SDM4	5′-GGCCGGAAGCTGCAGCAGGGAGGCC-3′
SDM5	5′-CCCTGCTACAGCTGCCGGCCGTCTGC-3′
SDM6	5′-GCAGACGGCCGGCAGCTGTAGCAGGG-3′
SDM7	5′-GGCCTCCCTTCTGCAGCTTCCGGCCGTCTG-3′
SDM8	5′-CAGACGGCCGGAAGCTGCAGAAGGGAGGCC-3′
SDM9	5′-CCCTTCTACAGCTGCCGGCCGTCTGCGGG-3′
SDM10	5′-CCCGCAGACGGCCGGCAGCTGTAGAAGGG-3′
SDM11	5′-CTCCTAGACCCGTCGCTGGCATGGAACGAG-3′
SDM12	5′-CTCGTTCCATGCCAGCGACGGGTCTAGGAG-3′
SDM13	5′-CGAGAACGGAAAGTGGCTATGCGGCTGCACCGTGGG-3′
SDM14	5′-GCTCTTGCCTTTCACCGATACGCCGACGTGGCACCC-3′
SDM15	5′-CCCAGTCAACGTCGCCTCATCTGATCTCACGGGC-3′
SDM16	5′-GCCCGTGAGATCAGATGAGGCGACGTTGACTGGG-3′
SDM17	5′-CGGGCCGACAAGATGCCTCTCGCATGTCCAC-3′
SDM18	5′-GTGGACATGCGAGAGGCATCTTGTCGGCCCG-3′
SDM19	5′-ATACCTCTCGCATGTCCGCCTCACAGAGGAGCAGGGACAT-3′
SDM20	5′-TATGGAGAGCGTACAGGCGGAGTGTCTCCTCGTCCCTGTA-3′

**Table 2:** Generated CK1δ-specific fragments, mutants and nomenclature of fusion proteins.

CK1δ fragment (aa)	mutant positions	primer pair	GST-CK1δ fusion protein	FP-#
Rat CK1δ (L07578, [77])				
305–428	–	P1/P2	GST-CK1δ^305–428^	FP1181
305–375	–	[74]	GST-CK1δ^305–375^	FP1006
305–375	S328A	SDM1/SDM2	GST-CK1δ^305–375 S328A^	FP1269
305–375	S328A, T329A	SMD3/SDM4	GST-CK1δ^305–375 S328A, T329A^	FP1359
305–375	S328A, S331A	SDM5/SDM6	GST-CK1δ^305–375 S328A, S331A^	FP1360
305–375	T329A	SDM7/SDM8	GST-CK1δ^305–375 T329A^	FP1344
305–375	S331A	SDM9/SDM10	GST-CK1δ^305–375 S331A^	FP1340
305–375	S361A	SDM11/SDM12	GST-CK1δ^305–375 S361A^	FP1295
305–375	S370A	SDM13/SDM14	GST-CK1δ^305–375 S370A^	FP1317
353–375	–	[74]	GST-CK1δ^353–375^	FP1022
353–375	S370A	[74]	GST-CK1δ^353–375 S370A^	FP1021
375–428	–	P3/P2	GST-CK1δ^375–428^	FP1183
375–428	S382A	SDM15/SDM16	GST-CK1δ^375–428 S382A^	FP1347
375–428	T392A	SDM17/SDM18	GST-CK1δ^375–428 T392A^	FP1322
375–428	T397A	SDM19/SDM20	GST-CK1δ^375–428 T397A^	FP1221
375–428	T427A	P3/P4	GST-CK1δ^375–428 T427A^	FP1262
1–428	–	[78]	GST-CK1δ	FP449
1–428	S328A	SDM1/SDM2	GST-CK1δ^S328A^	FP1267
1–428	S370A	[74]	GST-CK1δ^S370A^	FP1030
1–428	T397A	SDM17/SDM18	GST-CK1δ^T397A^	FP1184
1–428	S328A, S370A	SDM1/SDM2, SDM13/SDM14	GST-CK1δ^S328A, S370A^	FP1292
1–428	S328A, T397A	SDM1/SDM2, SDM19/SDM20	GST-CK1δ^S328A, T397A^	FP1268
1–428	S328A, S370A, T397A	SDM1/SDM2, SDM13/SDM14, SDM19/SDM20	GST-CK1δ^S328A, S370A, T397A^	FP1293
Human CK1δ TV1 (NM_001893.4)				
375–415	–	P5/P6	GST-CK1δ^375–415 TV1^	FP1341
Human CK1δ TV2 (NM_139062.2)				
375–409	–	P5/P7	GST-CK1δ^375–409 TV2^	FP1343

Phosphorylation site mutants of wild-type full length rat CK1δ and CK1δ C-terminal fragments were created by using the QuickChange site-directed mutagenesis kit (Agilent Technologies, USA) and the SDM primer pairs as indicated in tables 1 and 2.

The construction of pGEX-p53^1–64^ (FP267) encoding amino acids 1–64 of wild-type mouse p53 has been described elsewhere [78]. The expression vector pGEX-β-catenin^1–181^ (FP1355), coding for amino acids 1–181 of wild-type mouse β-catenin was generated according to [79].

### Overproduction and Purification of Glutathione S-transferase Fusion Proteins

The production and purification of glutathione S-transferase (GST) fusion proteins were carried out as described previously [61].

### Antibodies

For immunoprecipitation and the detection of total Chk1 in Western blot a mouse monoclonal anti-Chk1 antibody (IP: 2 µg; Western blot: 1∶750; C9358, Sigma-Aldrich, USA) was used. Phosphorylated Chk1 (pChk1^S345^) was detected using the rabbit monoclonal anti-phospho-Chk1 (Ser-345) antibody 133D3 (1∶500; 2348, Cell Signaling Technology, USA). Furthermore, the goat polyclonal anti-CK1δ antibody R-19 (Western blot: 1∶1000; sc-6474, Santa Cruz Biotechnology, USA), the mouse monoclonal anti-CK1δ antibody 128A (IP: 2 µg; ICOS Corporation, USA), and the mouse monoclonal anti-β-actin antibody AC-15 (Western blot: 1∶10000; A5441, Sigma-Aldrich, USA) were used. Secondary horseraddish peroxidase (HRP) conjugated antibodies for Western blot analyses were supplied by GE Healthcare, USA (anti-mouse HRP, NA931V, 1∶10000 and anti-rabbit HRP, NA934V, 1∶2000) and Rockland Immunochemicals, USA (anti-goat HRP, 605–4302, 1∶2500).

### In vitro Kinase Assays

*In vitro* kinase assays were carried out in kinase buffer (25 mM Tris-HCl pH 7.5, 10 mM MgCl_2_, 0.1 mM EDTA, 10 µM ATP) using 2 µCi [γ-^32^P]-ATP per reaction. Recombinant human Chk1 (27.5 ng/reaction; Invitrogen, USA), GST-CK1δ (wild-type or mutant, 15 ng/reaction), or single fractions of fractionated HT1080 cell extracts were used as source of enzyme and either GST-p53^1–64^ (FP267, [80]; 0.5 µg/reaction), α-casein (C7891; Sigma-Aldrich, USA; 5 to 0.16 µg/reaction), or several CK1δ C-terminal GST fusion proteins (see table 2; 0.5 µg/reaction) were used as substrates. In kinetic assays 6.5 ng of recombinant kinase was used per reaction. Reactions were incubated for 30 min at 30°C. Where indicated, the CK1δ- and ε-specific inhibitors D4476 [81], compound **17** [82], compound **8** (similar to the compounds published in [58]), or the Chk1-specific inhibitor SB-218078 were used in given concentrations. Proteins were separated by SDS-PAGE and bands containing [^32^P]-labeled proteins were visualized on dried gels by autoradiography. For quantitative analyses phosphorylated products were excised from Coomassie-stained gels and phosphate incorporation was measured by Cherenkov counting (LC6000IC, Beckman Coulter, USA). Results are shown as normalized graphs and are representative for measurements at least performed in triplicate. Error bars indicate the standard error of the mean. Data were normalized towards appropriate control measurements. Statistical analyses including the determination of the kinetic parameters V_max_ (maximum catalytic velocity) and K_m_ (Michaelis constant) as well as the 50% inhibitory concentration (IC_50_) for SB-218078 were performed using GraphPad Prism 5 (GraphPad Software, USA).

### Immunoprecipitation Kinase Assay

Using 2 µg of specific antibodies Chk1 or CK1δ were precipitated from 250 µg of HT1080 protein extracts after cells were treated with hydroxyurea. The phosphatase inhibitors sodium fluoride (50 mM) and sodium orthovanadate (0.2 mM) were added to the lysis buffer to protect proteins from getting dephosphorylated. Antibody complexes were captured using 30 µl of protein G sepharose (50% (v/v) protein G sepharose in PBS; GE Healthcare, USA). Sepharose-bound complexes were washed three times with lysis buffer and once with kinase buffer without ATP. Determination of Chk1- or CK1δ-speicific kinase activity was carried out in kinase buffer using 2 µCi [γ-^32^P]-ATP per reaction and either 1 µg CHKtide peptide substrate (Millipore, Billerica, USA) or 0.5 µg GST-p53^1–64^ (FP267) were used as substrate. Reactions were incubated for 30 min at 30°C and reactions using CHKtide peptide substrate were stopped by absorption of the complete reaction volume on P81 filter paper. Papers were washed three times with 75 mM orthophosphoric acid and once in acetone. Dried papers were used for Cherenkov counting to quantify phosphate incorporation. Reactions containing FP267 were separated by SDS-PAGE and phosphate incorporation into FP267 was determined by Cherenkov counting. For co-incubation experiments activated, precipitated Chk1 was incubated with GST-CK1δ for 10 min at 30°C prior to addition of GST-β-catenin^1–181^ (FP1355; 0.5 µg/reaction) and another incubation step at 30°C for 30 min.

### Two-dimensional Phosphopeptide Analysis

*In vitro* phosphorylated GST-CK1δ fusion proteins were analyzed by two-dimensional phosphopeptide analysis using a standard protocol described earlier [83]. Briefly, *in vitro* phosphorylated proteins were separated by SDS-PAGE and blotted onto a PVDF membrane (Hybond-P, GE Healthcare, UK). Membrane fragments containing labeled proteins were incubated in 100 mM acetic acid containing 5% (w/v) polyvinylpyrrolidone at 37°C for 30 min, extensively washed with 50 mM ammonium bicarbonate buffer, digested with TPCK-trypsin (Thermo Fisher Scientific, Germany), and oxidized with performic acid on ice for two hours. Phosphopeptides were analyzed on cellulose TLC plates (Merck, Germany) by electrophoresis at pH 1.9, followed by ascending chromatography in a buffer containing butanol/pyridine/acetic acid/H_2_O, 15∶10∶3∶12 (v/v).

### Phosphoamino Acid Analysis

Phosphoamino acid analysis was performed as described earlier [83]. Phosphopeptides were eluted from the TLC plate and subsequently hydrolyzed in 6 N HCl at 120°C. Resulting amino acids were mixed with phosphoamino acid standards (Sigma-Aldrich, USA) and analyzed by two-dimensional electrophoresis at pH 1.9 and 3.6. Phosphoamino acid positions were determined after staining of the standards with ninhydrin staining solution (0.2% (w/v) ninhydrin and 4% (v/v) acetic acid in acetone). [^32^P] labeled amino acids were visible after autoradiography.

### Nano LC-MS/MS Analysis of Purified GST-CK1δ305–428

Samples were loaded on a NuPAGE Bis-Tris 4%–12% (w/v) gradient gel (Invitrogen, USA). Then, Coomassie-stained GST-CK1δ^305–428^ bands were pooled and in-gel digested with trypsin and GluC, respectively, as described elsewhere [84].

10% of the digest was directly analyzed by LC-MS/MS, acetonitrile was added to 90% of the peptide mixture to a final concentration of 30% and the pH was adjusted to 2–3. Enrichment of phosphopeptides by titanium dioxide chromatography was done as described previously [85] with the following modifications: phosphopeptide elution from the beads was performed three times with 100 µl 40% (v/v) ammonium hydroxide solution in 60% (v/v) acetonitrile at a pH of >10.5.

Analyses of the peptides were done on a Proxeon Easy-LC system (Proxeon Biosystems, Denmark) coupled to a LTQ-Orbitrap-XL (Thermo Fisher Scientific, Germany) equipped with a nanoelectrospray ion source (Proxeon Biosystems, Denmark) as described previously [86]. The five most intense precursor ions were fragmented by activation of neutral loss ions at −98, −49, and −32.6 relative to the precursor ion (multistage activation). Mass spectra were analyzed using the software suite MaxQuant, version 1.0.14.3 [87]. The data were searched against a target-decoy *Escherichia coli* database containing 4163 forward protein sequences, the sequence of the GST-CK1δ^305–428^ fragment, and 262 frequently observed contaminants. Trypsin and GluC, respectively, were set as proteases in which two missed cleavage sites were allowed. Beside acetylation at the N-terminus and oxidation of methionine, phosphorylation of serine, threonine, and tyrosine were set as variable modifications. Carbamidomethylation of cysteine was set as fixed modification. Initial precursor mass tolerance was set to 7 parts per million (ppm) at the precursor ion and 0.5 Da at the fragment ion level. Spectra of modified peptides were manually validated. MASCOT scores are given for probabilistic evaluation of mass spectrometric results. Total obtained sequence coverage was 47.6%.

### Cell Lines, Treatment of Cells and Cell Lysis

HT1080 cells [76] were grown in Dulbecco’s modified Eagle’s medium (DMEM, Gibco, USA) supplemented with 10% (v/v) FCS, 100 units/ml penicillin, and 100 µg/ml streptomycin in a humidified 5% cabon dioxide atmosphere at 37°C. Where indicated cells were either untreated or treated with 2.5 mM hydroxyurea (Sigma-Aldrich, USA) and/or 10 µM SB-218078 (Tocris Bioscience, UK) for the indicated periods of time. Cells were then washed with ice cold PBS and lysed either in NP40 lysis buffer (50 mM Tris-HCl pH 8.0, 150 mM NaCl, 10% (v/v) glycerol, 5 mM DTT, 1 mM EDTA, 1 mM EGTA, and 25 µg/ml aprotinin) or in sucrose lysis buffer (20 mM Tris-HCl pH 7.0, 0.27 M sucrose, 1 mM EDTA, 1 mM EGTA, 1% (v/v) Triton X-100, 1 mM benzamidine, 25 µg/ml aprotinin). In each case cell lysates were cleared by centrifugation at 13000 rpm for 10 min at 4°C. Concentration of total protein in cleared lysates was determined using the BCA protein assay (Thermo Fisher Scientific, Germany).

### Immunoprecipitation

For immunoprecipitation 250 µg whole cell protein extract was diluted to a final volume of 500 µl and incubated with 2 µg of Chk1-specific antibody or control serum overnight under slow rotation at 4°C. Antibodies were captured with 40 µl protein G sepharose (50% (v/v) protein G sepharose in PBS) under continued rotation at 4°C for 5 hours. Immunocomplexes were collected by pulse centrifugation and washed three times with 250 µl NP40 lysis buffer. Finally, collected complexes were mixed with 2× SDS sample buffer and incubated for 10 min at 37°C and 750 rpm in a thermal mixer. All samples were pulse-centrifuged, the supernatant was used for SDS-PAGE and subsequent Western blotting.

### SDS-PAGE and Western Blot Analysis

Cell lysates used for Western blot analyses were prepared in NP40 lysis buffer. 80 µg of cleared extracts were separated on 12.5% (v/v) gels in SDS-PAGE and transferred to a PVDF membrane (Hybond-P, GE Healthcare, UK). For immunodetection of Chk1, pChk1^S345^, CK1δ, and β-actin membranes were probed with appropriate antibodies overnight. Immunocomplexes were detected using peroxidase conjugated anti-mouse or anti-rabbit IgG and chemiluminescence detection.

### Fractionation of Cell Extracts

For subsequent fractionation cells were lysed in sucrose lysis buffer. Cell lysates were passed through a 0.4 µm filter and equal amounts of total protein were applied to an anion exchange column (Resource Q, GE Healthcare, UK) attached to an Ettan LC purifier (GE Healthcare, UK). Bound proteins were eluted with a linear ascending gradient between 0 and 1000 mM NaCl in 50 mM Tris-HCl pH 7.5, 1 mM EDTA, 5% (v/v) glycerol, 0.04% (v/v) Brij, 1 mM benzamidine, 25 µg/ml aprotinin, and 0.1% (v/v) β-mercaptoethanol.

## Results

### CK1δ is Phosphorylated by Chk1 *in vitro*

**Figure 1:**
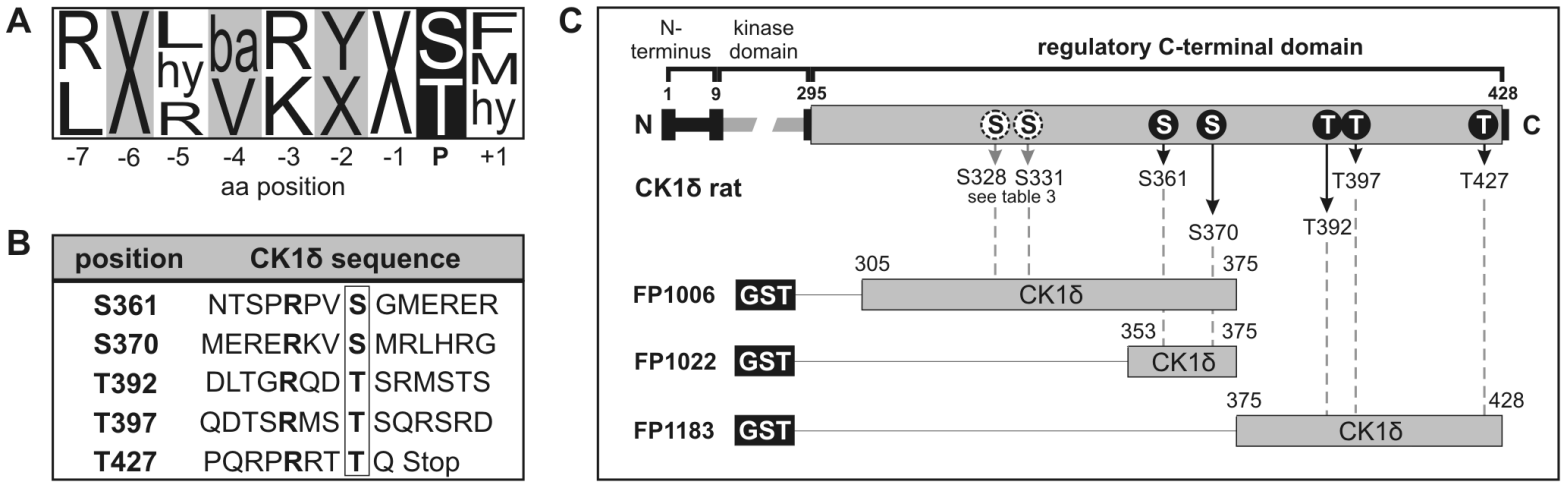
The CK1δ C-terminal domain contains target sites for Chk1. (**A**) Consensus motif for Chk1 as suggested by O’Neill and co-workers [75]. X, no particular amino acid preference; hy, hydrophobic amino acid; ba, basic amino acid. (**B**) Surrounding sequences of potential phosphorylation sites for Chk1 within the C-terminal domain of CK1δ, determined according to the published consensus sequence [75]. (**C**) The wild-type GST-CK1δ fusion proteins FP1006, FP1022, and FP1183 were generated according to the positions of the predicted Chk1 phosphorylation sites within the C-terminal domain of rat CK1δ.

Phosphorylation of the C-terminal regulatory domain of CK1δ by inhibitory autocatalysis or cellular kinases plays an important role in regulating CK1δ kinase activity. We previously reported that several cellular kinases are able to phosphorylate this C-terminal region and that phosphorylation of CK1δ by PKA at Ser-370 significantly affects CK1δ activity [74]. This site is also a potential target for phosphorylation by Chk1. Since both kinases, CK1δ and Chk1, are involved in regulating cell cycle progression and p53 functions, the present study intends to demonstrate a possible interaction between Chk1 and CK1δ. According to the Chk1 consensus motif published by O’Neill and colleagues [75], the C-terminal regulatory domain of CK1δ contains several putative target sites for Chk1-mediated phosphorylation (figure 1A and B). Initially, mass spectrometric examination of trypsin or GluC digested GST-CK1δ^305–428^ (FP1181), which was phosphorylated by Chk1 *in vitro*, revealed that CK1δ can be phosphorylated by Chk1 at positions Ser-328, Thr-329, Ser-331, Ser-361, and Ser-382 (table 3). However, regarding the weaker results for Ser-361 and Ser-382, phosphorylation of these sites has to be confirmed by further methods.

**Table 3:** Nano LC-MS/MS analysis of purified GST-CK1δ^305–428^.

site position	modified sequence	peptide range	intensity	MASCOT score
S328	GLPS(ph)TASGR	325–333	874860	14.16
T329	GLPST(ph)ASGR	325–333	67401	14.16
S331	GLPSTAS(ph)GR	325–333	9052800	14.16
S361	PVS(ph)GM(ox)ER	359–365	25040	23.79
S382	GAPVNVS(ph)SSDLTGR	376–389	42918	42.51

Phosphate-labeled GST-CK1δ^305–428^ was isolated and digested by trypsin. The resulting phosphopeptides were analyzed by Nano LC-MS/MS analysis as described in Materials and Methods. Mass spectra were analyzed using the software suite MaxQuant, version 1.0.14.3. Potential phosphorylated peptides and residues matching the sequence of rat CK1δ are shown. Ph, phosphorylation; ox, oxidized.

**Figure 2:**
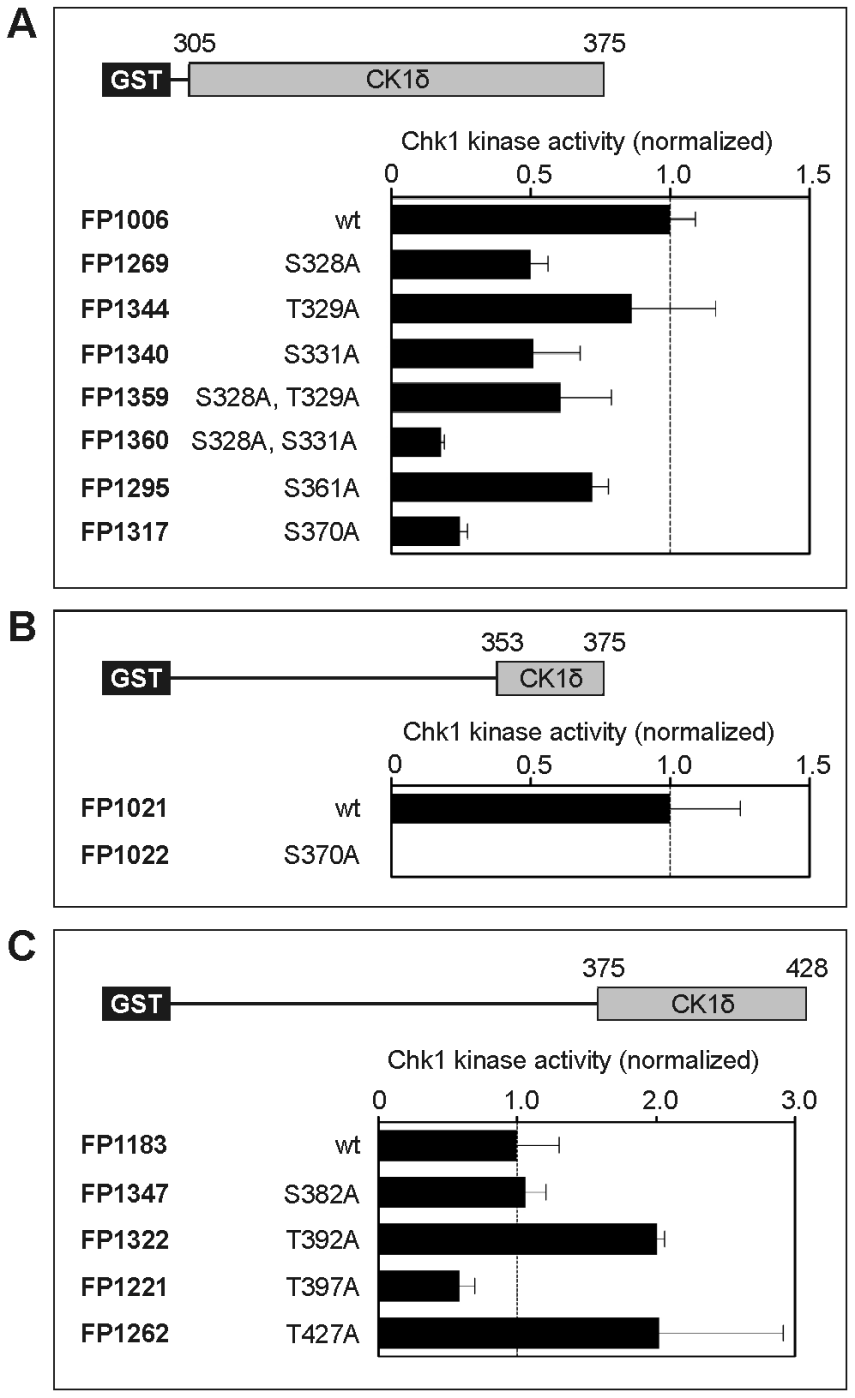
CK1δ is phosphorylated by Chk1 *in vitro.* Chk1-mediated phosphorylation of three C-terminal CK1δ fusion protein sets containing either wild-type or mutant sequences encompassing amino acids 305–375 (**A**), 353–375 (**B**), and 375–428 (**C**) of the rat CK1δ sequence. The GST-CK1δ fusion proteins were phosphorylated by Chk1 *in vitro* and separated in SDS-PAGE. Substrate phosphorylation was quantified by Cherenkov counting. Results are shown as normalized bar graphs.

In order to validate the potential phosphorylation sites predicted by the consensus motif or detected by the mass spectrometric analysis three sets of fusion proteins encompassing amino acids 305–375, 353–375, and 375–428 of the rat CK1δ sequence were designed and created by using the templates and primers given in tables 1 and 2. Each fusion protein sequence contained potential Chk1 phosphorylation sites (figure 1B and C). Mutations of the respective positions were introduced into the wild-type CK1δ fragments of each set of fusion proteins in order to detect residues which can be phosphorylated by Chk1 *in vitro* (see figures 1C and 2). In order to eliminate the effects of intramolecular autophosphorylation, all fragments were created without containing any parts of the CK1δ kinase domain. Quantification of Chk1-mediated phosphorylation of the fusion proteins (wild-type vs. mutant) in each set provided strong evidence for phosphorylation of Ser-328, Ser-331, Ser-370, and Thr-397 while data for Thr-329 and Ser-361 are less striking. Phosphorylation of Ser-382, Thr-392, and Thr-427 could not be verified by these experiments (figure 2).

**Figure 3:**
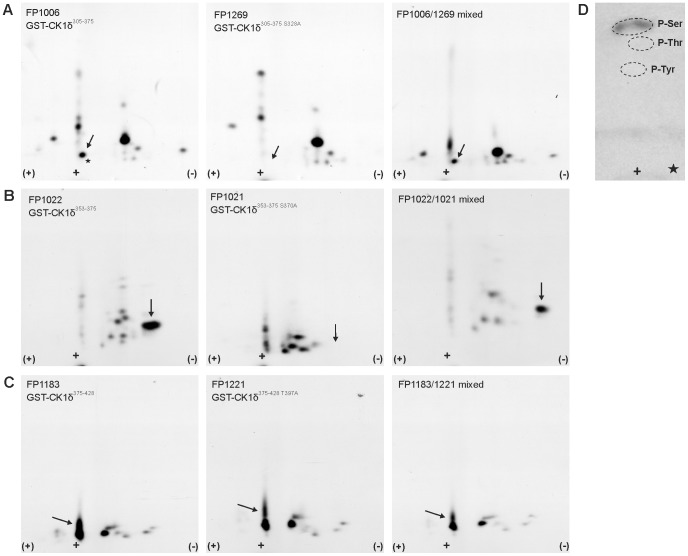
Phosphopeptide and phosphoamino acid analyses of Chk1-phosphorylated GST-CK1δ fusion proteins. Fusion proteins GST-CK1δ^305–375^ (FP1006) and GST-CK1δ^305–375 S328A^ (FP1269) (**A**); GST-CK1δ^353–375^ (FP1022) and GST-CK1δ^353–375 S370A^ (FP1021) (**B**); GST-CK1δ^375–428^ (FP1183) and GST-CK1δ^375–428 T397A^ (FP1221) (**C**) were phosphorylated by Chk1 *in vitro*, processed and analyzed by two-dimensional phosphopeptide analyses as described in the Materials and Methods section. Arrow positions indicate identical phosphopeptide positions. Subsequent phosphoamino acid analysis of the indicated peptide from (A) is shown in panel (**D**). Mixed analyses confirm the identity of the arrow-marked peptides.

Additionally, the significance of these findings was improved by phosphopeptide analyses using the GST-wt CK1δ fusion proteins GST-CK1δ^305–375^ (FP1006), GST-CK1δ^353–375^ (FP1022), and GST-CK1δ^375–428^ (FP1183) as well as corresponding GST-mutant CK1δ proteins. Expectedly, one phosphopeptide is missing in the phosphopeptide analysis of GST-CK1δ^305–375 S328A^ (FP1269) compared to that of GST-CK1δ^305–375^ (FP1006) (figure 3A). Phosphoamino acid analysis of this peptide clearly confirms the presence of phospho-serine (figure 3D). No new insights could be gained from the phosphopeptide patterns of GST-CK1δ^305–375 T329A^ (FP1344), GST-CK1δ^305–375 S331A^ (FP1340), and GST-CK1δ^305–375 S361A^ (FP1295) (data not shown). Phosphorylation of Ser-370 could clearly be verified by phosphopeptide analysis of GST-CK1δ^353–375 S370A^ (FP1021) which compared to GST-CK1δ^353–375^ (FP1022) is lacking one phosphopeptide most probably due to mutation of Ser-370 in FP1021 (figure 3B). Comparison of the phosphopeptide pattern of GST-CK1δ^375–428 T397A^ (FP1221) with that of GST-CK1δ^375–428^ (FP1183) revealed a weakened signal at the indicated position, thereby confirming that Thr-397 can be phosphorylated by Chk1 *in vitro* (figure 3C). Phosphorylation patterns of GST-CK1δ^375–428 S382A^ (FP1347), GST-CK1δ^375–428 T392A^ (FP1322), and GST-CK1δ^375–428 T427A^ (FP1262) could not give any new information compared to GST-CK1δ^375–428^ (data not shown).

In conclusion, combined data of mass spectrometric and classical biochemical approaches provide evidence for the CK1δ C-terminal residues Ser-328, Ser-331, Ser-370, and Thr-397 to be targeted by Chk1 *in vitro*.

### Chk1-mediated Phosphorylation of CK1δ Shows Species-specific Differences

**Figure 4:**
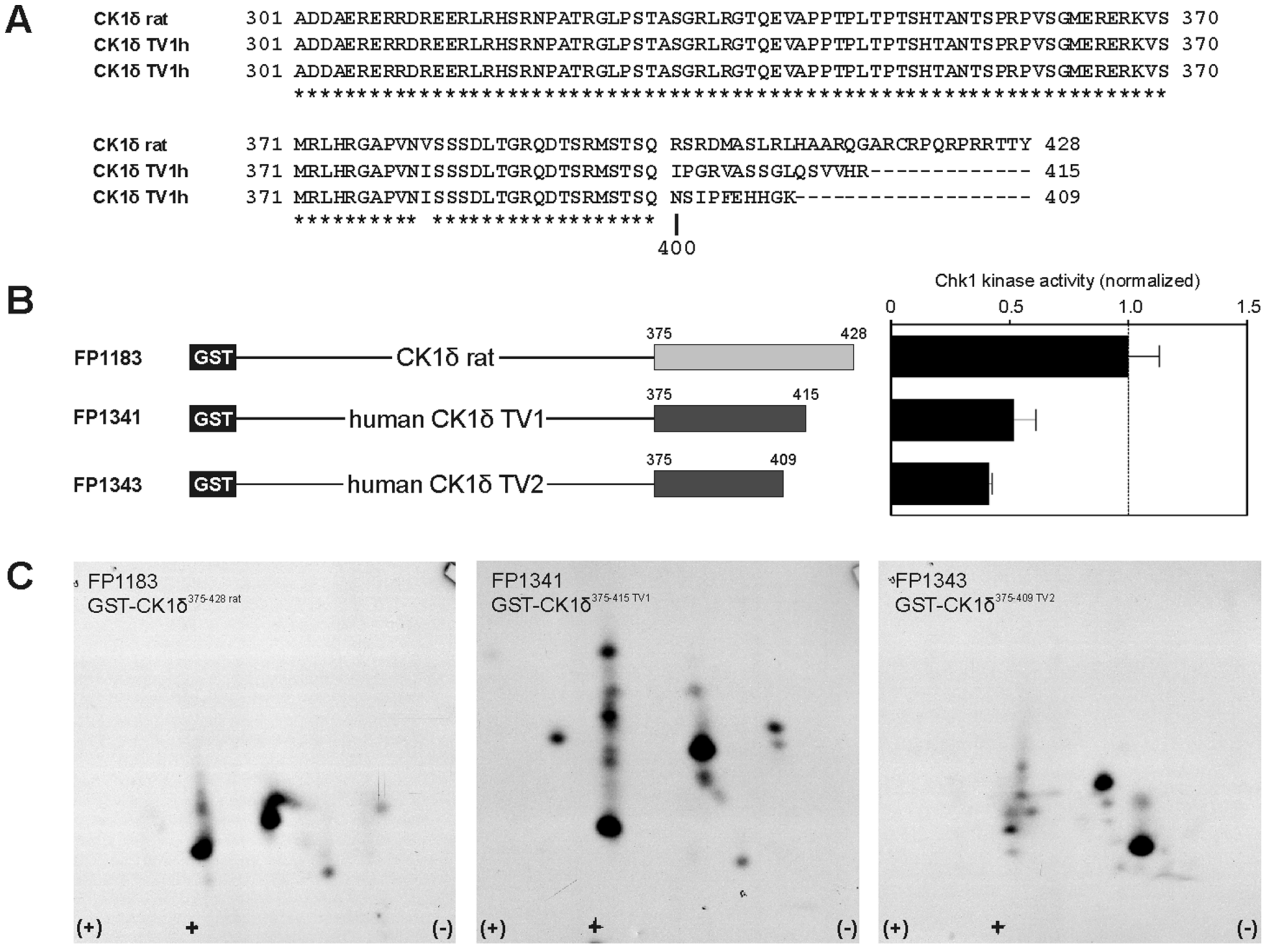
CK1δ shows species-specific differences in its sequences and phosphorylation by Chk1. (**A**) Alignment of the rat CK1δ sequence with the human CK1δ transcription variants (TV) 1 and 2. (**B**) GST-CK1δ^375–428 rat^ (FP1183), GST-CK1δ^375–415 TV1^ (human, FP1341), and GST-CK1δ^375–409 TV2^ (human, FP1343) were phosphorylated by Chk1 *in vitro*. The phosphorylated proteins were separated by SDS-PAGE and Chk1-mediated phosphorylation was quantified by Cherenkov counting of phosphorylated substrate bands. Results are shown as normalized bar graph. (**C**) GST-CK1δ^375–428 rat^ (FP1183), GST-CK1δ^375–415 TV1^ (FP1341), and GST-CK1δ^375–409 TV2^ (FP1343) were phosphorylated by Chk1 *in vitro* and processed for two-dimensional phosphopeptide analyses as described in the Materials and Methods section.

The GST-CK1δ fusion proteins used in the previous section all originate from the rat CK1δ sequence (FP449, [78]). However, when we try to apply the described results to human CK1δ isoforms, we have to pay attention on the different C-terminal sequences. Up to amino acid position 399, the two human CK1δ transcription variants are identical to the rat CK1δ sequence except one exchange from isoleucine to valine at position 381. From amino acid (aa) 400 the sequences differ significantly (figure 4A). As a consequence, C-terminal GST-CK1δ fusion proteins for both human CK1δ transcription variants starting from aa 375 were constructed and phosphorylation intensity and phosphorylation patterns of these GST-CK1δ fusion proteins upon Chk1-mediated phosphorylation were compared to that of rat CK1δ. The intensity of phosphorylation by Chk1 is decreased for CK1δ fusion proteins of human origin (FP1341 and FP1343) compared to that of the GST-rat CK1δ fusion protein FP1183 (figure 4B). Likewise, the phosphopeptide patterns of the analyzed variants are quite different (figure 4C). However, further analyses are needed to determine the phosphorylated positions and to describe the functional differences between these CK1δ variants.

### Chk1-targeted Phosphorylation Sites Play an Important Role in Modulating CK1δ Kinase Activity *in vitro*

**Figure 5:**
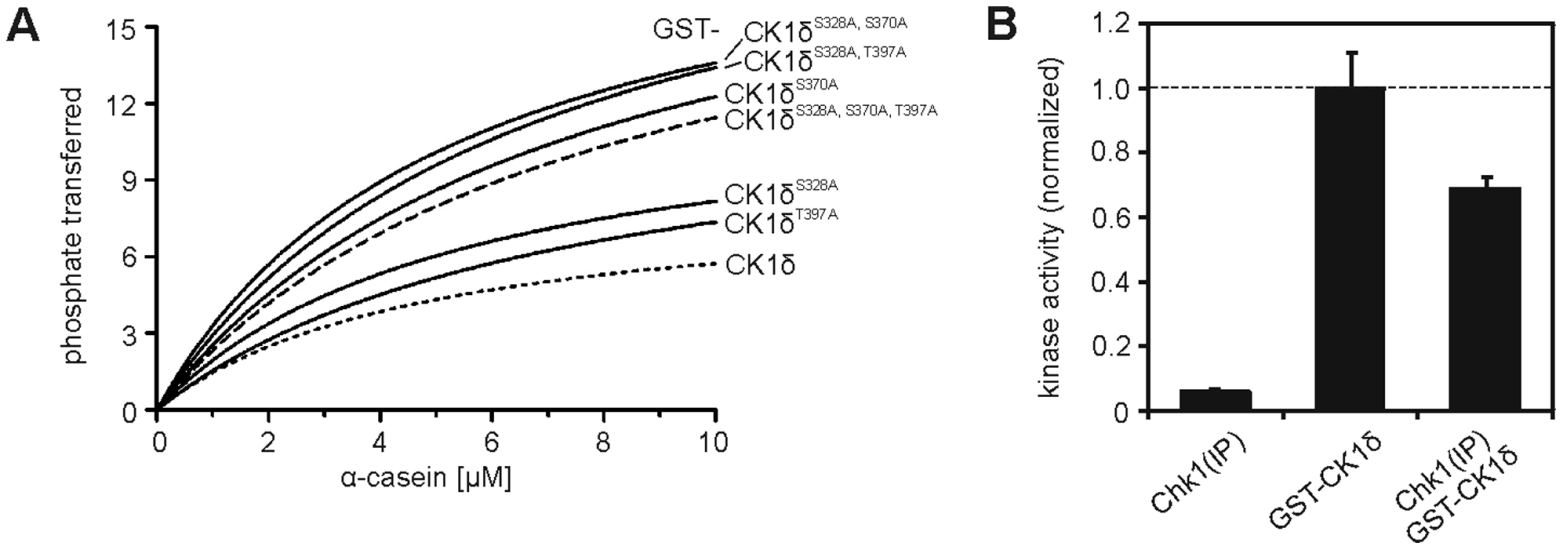
Chk1 target residues influence activity and kinetic parameters of CK1δ. (**A**) The kinetic parameters K_m_ and V_max_ of GST-wt CK1δ (FP449) and generated phosphorylation-site mutants were determined by *in vitro* kinase assays using α-casein as substrate. Substrate phosphorylation was quantified by Cherenkov counting and data were fitted to the Michaelis-Menten equation. V_max_ is expressed as pmol phosphate transferred per minute per mg of recombinant kinase. (**B**) GST-CK1δ was pre-incubated with activated Chk1 which was precipitated from hydroxyurea-treated HT1080 cells (Chk1(IP)) for 10 min. Subsequently, GST-β-catenin^1–181^ was phosphorylated by GST-CK1δ alone or after pre-incubation with Chk1(IP) for additional 30 min. Data are presented as normalized bar graph.

In order to investigate the importance of the so far identified target sites for Chk1 within the C-terminal domain of CK1δ, residues in GST-CK1δ (FP449) which have been identified as targets for Chk1-mediated phosphorylation were exchanged to alanine by site-directed mutagenesis and the kinetic parameters of wild-type CK1δ and the resulting CK1δ single and double phosphorylation-site mutants or the triple mutant were experimentally determined using α-casein as substrate. Data shown in figure 5A and table 4 clearly show that both maximum velocity (V_max_) and K_m_ values of all analyzed phosphorylation-site mutants increased compared to those of wild-type GST-CK1δ. GST-CK1δ^S370A^ and GST-CK1δ^S328A, S370A, T397A^ presented the highest K_m_ values in this test (7.378 and 7.739 µM, respectively), indicating lowest substrate affinity. Among the tested single-site mutants the most significant change in V_max_ could be obtained for GST-CK1δ^S370A^.

**Table 4:** Kinetic parameters of wild-type and mutant GST-CK1δ fusion proteins.

CK1δ mutant	V_max_	K_m_ [µM]
wild-type	8.506 (±0.493)	4.843 (±0.590)
S328A	12.660 (±0.767)	5.487 (±0.668)
S370A	21.320 (±1.676)	7.378 (±1.025)
T397A	12.660 (±1.467)	7.200 (±1.528)
S328A, S370A	20.810 (±1.461)	5.312 (±0.723)
S328A, T397A	22.300 (±1.699)	6.630 (±0.925)
S328A, S370A, T397A	20.310 (±1.802)	7.739 (±1.227)

Identified Chk1 target residues in GST-wt CK1δ (FP449) were exchanged to alanine and the kinetic parameters of the resulting mutants were determined using α-casein as substrate. Data were fitted to the Michaelis-Menten equation. Obtained V_max_ and K_m_ values are reported in table 4. V_max_ is expressed as pmol phosphate transferred per minute per mg of recombinant kinase. Standard error values are indicated in parentheses.

The relevance of the identified target sites for CK1δ kinase activity was furthermore indicated by co-incubating full length wild-type CK1δ with activated, precipitated Chk1 (Chk1(IP)) prior to addition of the non-canonical CK1δ substrate GST-β-catenin^1–181^ (FP1355). Chk1(IP) alone shows no notable phosphorylation of this substrate. In contrast, GST-β-catenin^1–181^ can be intensely phosphorylated by GST-CK1δ. When GST-CK1δ is pre-incubated with Chk1(IP) the GST-CK1δ-mediated phosphorylation of GST-β-catenin^1–181^ is markedly decreased by about 30% (figure 5B). However, this difference did not achieve statistical significance (p = 0.0565).

### CK1δ Phosphorylation State Influences the Effects of CK1δ-specific Inhibitors

**Figure 6:**
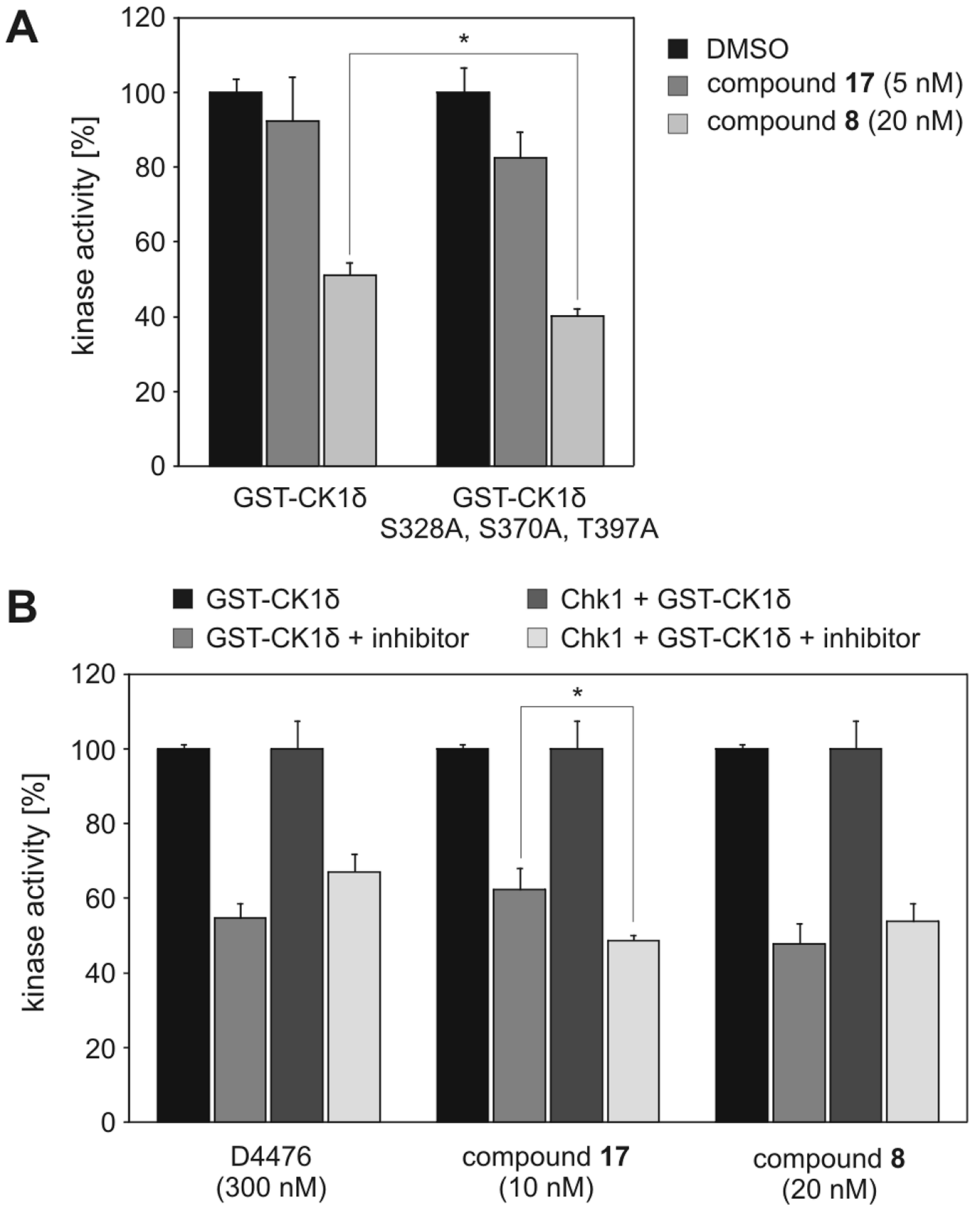
CK1δ phosphorylation state influences the effects of CK1δ- and ε-specific inhibitors. (**A**) Kinase assays were performed in the presence or absence of either 5 nM of compound **17** [82] or 20 nM of compound **8** [58] using GST-p53^1–64^ (FP267) as substrate and GST-wt CK1δ or GST-CK1δ^S328A, S370A, T397A^ as enzymes. * Observed effects are significant at p<0.05. (**B**) Kinase assays were performed in the presence or absence of either D4476 (300 nM), compound **17** (10 nM) or compound **8** (20 nM) using GST-p53^1–64^ (FP267) as substrate and GST-wt CK1δ alone or in combination with Chk1 as enzymes. * Observed effects are significant at p<0.05.

Because site-specific phosphorylation of CK1δ has been shown to influence the effects of CK1-specific inhibitors [58], [59] kinase assays were performed in the presence or absence of either 5 nM of compound **17** [82] or 20 nM of compound **8** [58] using GST-p53^1–64^ (FP267) as substrate and GST-wt CK1δ or GST-CK1δ^S328A, S370A, T397A^ as enzymes. Both wild-type and mutant GST-CK1δ were inhibited to a similar extend by compounds **17** and **8**. However, slightly stronger inhibitory effects for both small molecules could be detected for GST-CK1δ^S328A, S370A, T397A^ (figure 6A) with compound **8** even resulting in a significant difference.

Next we analyzed if Chk1-mediated phosphorylation of GST-CK1δ modulates the effects of the CK1δ- and ε-specific inhibitors D4476 [81], compound **17** [82] and compound **8** [58]. For this purpose kinase assays were performed in the presence or absence of either D4476 (300 nM), compound **17** (10 nM), or compound **8** (20 nM) using GST-p53^1–64^ (FP267) as substrate and GST-wt CK1δ alone or in combination with Chk1 as enzymes. Whereas Chk1-mediated phosphorylation of CK1δ significantly enhanced the ability of compound **17** to inhibit CK1δ kinase activity, the efficacy of D4476 was decreased in reactions containing Chk1 and CK1δ. The ability of compound **8** to inhibit CK1δ was only modestly affected in the presence of Chk1 (figure 6B) and did not lead to a statistically significant difference.

### CK1δ co-precipitates with Chk1 from HT1080 Protein Extracts

**Figure 7:**
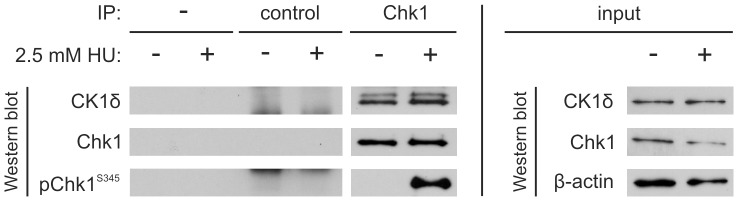
Co-immunoprecipitation of CK1δ with Chk1. Chk1 was precipitated from 250 µg of extracts from untreated or hydroxyurea-treated (2.5 mM, 2 h) HT1080 cells using 2 µg of a Chk1-specific antibody. Immunoprecipitation (IP) of Chk1 and co-precipitation of CK1δ was detected by Western blot. Experiments using non-specific serum (control) or no antibody at all served as negative controls. Detection of β-actin in the lysate input served as loading control.

In order to analyze the relevance of the Chk1/CK1δ interplay in cultured cells, the fibrosarcoma cell line HT1080 was treated with hydroxyurea (HU) to activate Chk1 [88]. As a first demonstration of a functional connection between Chk1 and CK1δ a Chk1-specific antibody was used to precipitate Chk1 from protein extracts prepared from HT1080 cells which were either untreated or treated with 2.5 mM HU for 2 h. Precipitated protein was separated in SDS-PAGE and used for Western blotting. The detection of CK1δ showed successful co-precipitation of CK1δ with Chk1 (figure 7). Specificity of binding was demonstrated by including a no-antibody control as well as a non-specific control. Precipitation of Chk1 was confirmed using antibodies against total Chk1 and activated Chk1 (pChk1^S345^). According to Walker and co-workers [89] phosphorylation of Ser-345 is mandatory for the activation of Chk1 after DNA damage and replication arrest. Expectedly, activated Chk1 could only be detected and precipitated after HU-treatment of HT1080 cells.

### Activation of Chk1 Negatively Affects CK1δ Kinase Activity in HT1080 Cells

**Figure 8:**
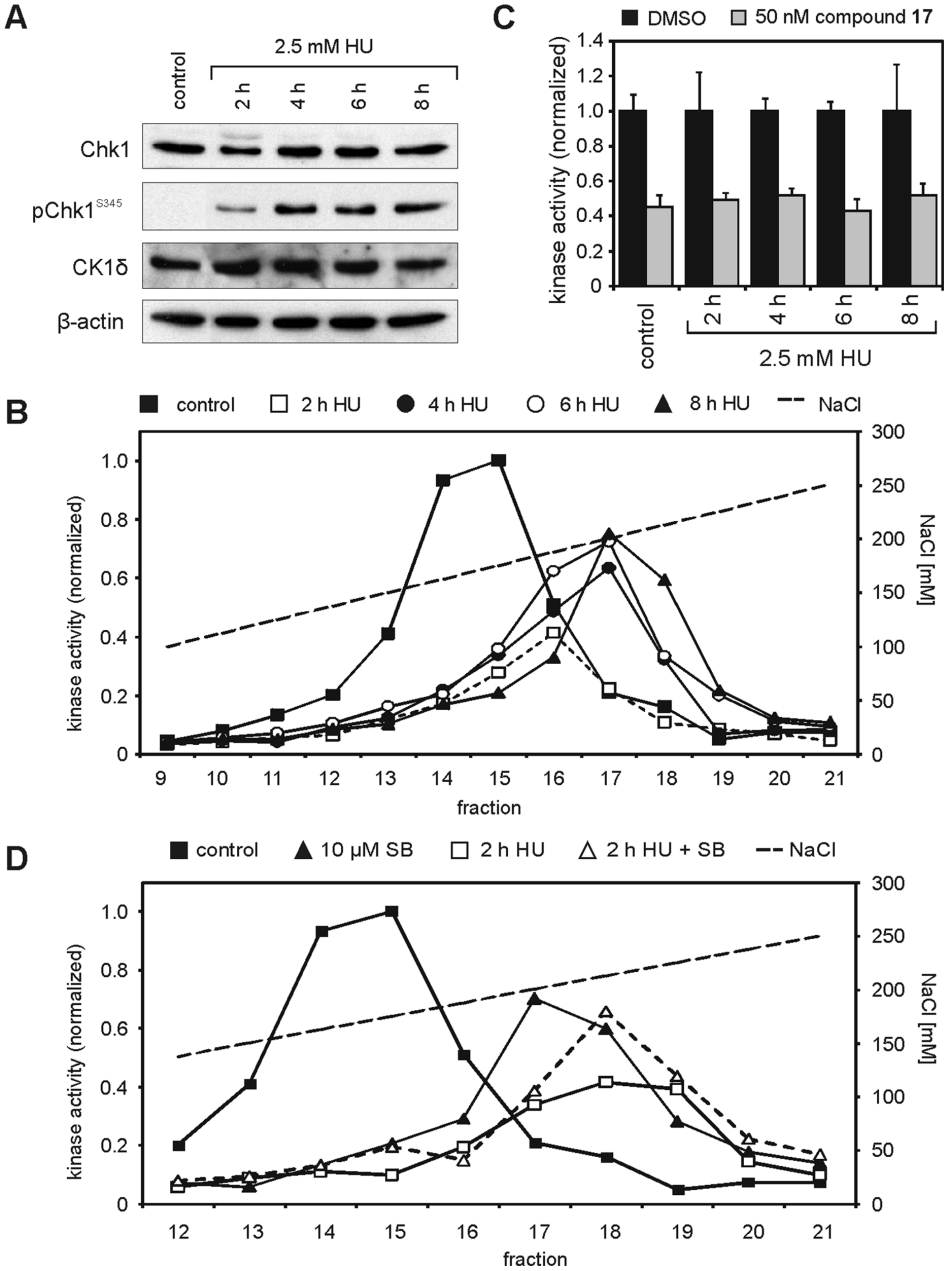
CK1δ-specific kinase activity in HT1080 cells is decreased after activation of Chk1. Cellular Chk1 was activated by treating HT1080 cells with 2.5 mM hydroxyurea (HU) for the indicated periods of time. (**A**) Activation of Chk1 (indicated by phosphorylated Ser-345) and expression levels of Chk1 and CK1δ were determined by immunoblotting. Detection of β-actin served as loading control. (**B**) CK1 kinase activity in fractionated extracts from HT1080 cells before and after treatment with 2.5 mM HU for 2, 4, 6 and 8 hours, respectively, was determined using GST-p53^1–64^ (FP267) as substrate. The detected kinase activity was normalized towards the untreated control. (**C**) Presence of CK1δ in the kinase peak fractions shown in (B) was confirmed by use of the CK1δ-specific inhibitor compound **17** at 50 nM [82]. (**D**) HT1080 cells were treated with 2.5 mM HU and/or the Chk1-specific inhibitor SB-218078 for 2 h. CK1 kinase activity in fractionated extracts was determined using GST-p53^1–64^ (FP267) as substrate. The detected kinase activity was normalized towards the untreated control.

Cellular Chk1 was activated by treating HT1080 cells with 2.5 mM HU for 2, 4, 6, or 8 h prior to the preparation of protein extracts. Control cells remained untreated. Activation of Chk1 was confirmed by detection of Ser-345 phosphorylation. Western blot analysis revealed that Ser-345 phosphorylation of Chk1 only emerged when HT1080 cells were treated with 2.5 mM HU, reaching a maximum pChk1^S345^ level after four hours of treatment (figure 8A). Expression levels of total Chk1 and CK1δ remained nearly stable over the total time of the experiment. However, *in vitro* co-incubation of Chk1 and CK1δ already demonstrated reduced activity of CK1δ in reactions also containing Chk1 (see figure 5B). Activated cellular Chk1 could phosphorylate and thereby modulate CK1δ activity in the same way. In order to examine this regulatory interaction in cultured cells, fractionated extracts of untreated and HU-treated cells were analyzed for CK1-specific kinase activity using GST-p53^1–64^ (FP267) as substrate for *in vitro* kinase reactions. Interestingly, after two hours of HU treatment CK1-specific kinase activity dramatically decreased by more than 60% compared to that of control cells (figure 8B). At later time points, kinase activity again increased but after eight hours still exhibited only 75% of the activity detected in control extracts. Since the kinase activity in the activity peak fractions was inhibited by the CK1δ-specific inhibitor compound **17** [82], the detected activity could clearly be assigned to the kinase activity of CK1δ (figure 8C). Furthermore, similar changes in cellular CK1δ and Chk1 activities could be observed in immunoprecipitation kinase assays using Chk1- and CK1δ-specific antibodies and specific peptide substrates (supporting figure S1).

Verifying the interaction of Chk1 and CK1δ within the cell, HT1080 cells were simultaneously exposed to Chk1-activating HU and the Chk1-specific inhibitor SB-218078 (10 µM; [90]) for two hours. Being compared to cells which were only treated with 2.5 mM HU the combined treatment with HU and SB-218078 showed higher CK1δ-specific kinase activity possibly caused by inhibition of Chk1-mediated phosphorylation of CK1δ (figure 8D). However, treatment with 10 µM SB-218078 alone also already resulted in a decrease of CK1δ-specific kinase activity (70% of control). This finding could be explained by an inhibitory effect on CK1δ which could be observed at increased concentrations of SB-218078 in *in vitro* kinase reactions (supporting figure S2).

## Discussion

CK1δ, a key regulator of various cellular processes and an interesting new drug target, is tightly regulated on various levels ([1] and references therein). On protein level reversible phosphorylation not only has a great impact on the activity of CK1δ [70]–[72], [74], it also modulates the effects of CK1-specific small molecule inhibitors [58], [59]. The C-terminal regulatory domain of CK1δ harbors phosphorylation sites, which can be targeted by several cellular kinases. Here, we provide evidence for the first time that Chk1 is one of these kinases specifically phosphorylating rat CK1δ within the C-terminal domain on Ser-328, Ser-331, Ser-370, and Thr-397. Furthermore, we show that CK1δ and Chk1 are cellular interaction partners.

The use of state of the art mass spectrometry, CK1δ phosphorylation site mutants, and classical biochemical methods revealed that Chk1 phosphorylates Ser-328, Ser-331, Ser-370, and Thr-397 within the C-terminal domain of rat CK1δ. These findings highlight the central role of Ser-370, which can be phosphorylated by a number of cellular kinases, including PKA, AKT, Clk2, and PKC-α [74]. Our results also show that not all sites containing the Chk1 consensus motif suggested by O’Neill and co-workers [75] are phosphorylated by Chk1 *in vitro*. Furthermore, several Chk1-targeted posphorylation sites detected by mass spectrometry could not be verified in other *in vitro* experiments and vice versa. Ser-370 and Thr-397 were only identified by the use of phosphorylation-site mutants in kinase reactions and two-dimensional phosphopeptide analyses highlighting the potential of these two methods. The failure to detect phosphorylation of these sites by mass spectrometry could have several reasons: (i) peptides resulting from tryptic digestion (Ser-370: VSMR; Thr-397: MSTSQR) are too small to be resolved by Nano LC-MS/MS, (ii) GST-CK1δ^305–428^ used as substrate was incompletely phosphorylated, or (iii) loss of phosphorylation at these sites occurred during sample processing. Additional analysis of phosphopeptides derived from GluC digestion of GST-CK1δ^305–428^ also failed in confirming phosphorylation of Ser-370 and Thr-397 but revealed that aa 340 to 366 did not contain any modified residue. This is in line with our phosphorylation analyses showing that Chk1 does not phosphorylate any amino acid of CK1δ between Ser-332 and Ser-369.

Since shorter CK1δ phosphopeptides like those containing Ser-370 and Thr-397 are likely to escape detection by Nano LC/ES-MS/MS, data from mass spectrometry should always be reviewed carefully and confirmed by alternative methods. This escape from detection could also explain why so far none of the large-scale mass spectrometry studies has shown *in vivo* phosphorylation of CK1δ at Ser-370, a site clearly identified as target for several cellular kinases using classical methods ([74] and this manuscript).

Although Ser-328 could be identified as a target for Chk1 by all of the employed experimental approaches, the entirety of *in vitro* data could not clearly point out the role of Ser-331. On the one hand Chk1-mediated phosphorylation of GST-CK1δ^305–375^ by Chk1 was decreased when Ser-331 is mutated to alanine and mutation of both, Ser-328 and Ser-331, is able to decrease phosphorylation intensity even below the levels detected for the respective single-site mutants. Also mass spectrometric data strongly suggest Ser-331 to be phosphorylated by Chk1. On the other hand, the performed phosphopeptide analyses could not clearly confirm phosphorylation of Ser-331 by Chk1 when GST-CK1δ^305–375 S331A^ (FP1340) was compared to GST-CK1δ^305–375^ (FP1006) (data not shown). Therefore this site only was considered as phosphorylation target with pending validation.

We furthermore provide evidence that in addition to rat CK1δ also human CK1δ TV1 and 2 are phosphorylated by Chk1. Up to aa 399 all three proteins are identical and our *in vitro* data for rat CK1δ can easily be applied to the human CK1δ TV1 and 2 as well. Beyond aa 399 the primary sequences of human CK1δ TV1 and 2 differ significantly from the rat sequence but also contain residues, which can be phosphorylated by cellular kinases [74], [91]–[99]. Therefore, additional analyses are necessary to identify all Chk1-targeted phosphorylation sites in the GST-human CK1δ C-terminal fusion proteins GST-CK1δ^375–415 TV1^ and GST-CK1δ^375–409 TV2^.

In order to examine the importance of Ser-328, Ser-370, and Thr-397 for the catalytic activity of CK1δ *in vitro,* the kinetic constants of the phosphorylation of α-casein by GST-CK1δ^S328A^, GST-CK1δ^S370A^, GST-CK1δ^T397A^, GST-CK1δ^S328A, S370A^, GST-CK1δ^S328A, T397A^, and GST-CK1δ^S328A, S370A, T397A^ were calculated experimentally. Especially GST-CK1δ^S370A^ and the tested multi-site mutants (GST-CK1δ^S328A, S370A^, GST-CK1δ^S328A, T397A^, and GST-CK1δ^S328A, S370A, T397A^) differed remarkably in their kinetic parameters compared to those of wild-type CK1δ. The maximum catalytic velocity, as expressed by V_max_, is nearly doubled for these mutants. However, the K_m_ values are also increasing, indicating decreased substrate affinity for these CK1δ mutants. These findings illustrate a major influence of Ser-370 and Thr-397 on CK1δ activity. The potential phosphosite Ser-331 was not taken into account in these experiments since its exact role has not finally been validated yet.

In a first approach to establish a direct functional connection between Chk1 and CK1δ, phosphorylation of the non-canonical substrate GST-β-catenin^1–181^ [79] was successfully decreased by co-incubating GST-CK1δ with precipitated, activated Chk1. GST-β-catenin^1–181^ in contrast to GST-p53^1–64^ cannot be phosphorylated by Chk1 and the use of activated Chk1 furthermore allows to compare this result to previously published data generated using the active subunit of PKA [74]. Although Chk1 shows basal activity it can be activated five to ten-fold by phosphorylation of Ser-345 by upstream kinases (e.g. ATR) (reviewed by [100]). As published by Giamas and colleagues [74] the activity of GST-CK1δ is reduced by almost 80% when being subjected to phosphorylation by the active subunit of PKA. Results obtained for the effects of activated Chk1 on CK1δ kinase activity are in line with these data. However, the influence of Chk1 seems to be weaker since the observed reduction in GST-CK1δ-specific activity in presence of Chk1 only accounts to approximately 30%.

In further analyses we demonstrate that mutation of Chk1 target sites in GST-CK1δ as well as co-incubation of recombinant Chk1 and GST-CK1δ results in modification of the effects of CK1-specific inhibitors. These differences are specific for the inhibition of CK1δ because selectivity screening approaches revealed that the tested classes of inhibitors did not show any effects on the activity of Chk1 [58], [82]. This finding is consistent with previous work reporting the impact of site-specific phosphorylation on inhibitor efficacy [58], [59]. However, these effects are specifically dependent on the respective inhibitor molecule and its binding mode.

In addition we provide evidence that Chk1 influences CK1δ activity in the cellular context. Results from co-immunoprecipitation and co-incubation experiments lead to the assumption, that a regulatory interaction between CK1δ and Chk1 might also be observed *in vivo*. Actually, CK1δ activity in HT1080 cells dramatically decreases after hydroxyurea treatment, which induces the depletion of dNTP pools resulting in S-phase arrest and Chk1 activation [88], [101]. Simultaneous treatment with the Chk1-specific inhibitor SB-218078 partly reverses the HU-induced effects on cellular CK1δ kinase activity. Here however, we also have to take into account the inhibitory effects of SB-218078 on CK1δ activity we detected *in vitro*, which may falsify the presented result. Without this effect SB-218078 may potentially lead to complete reversal of the HU-induced effects.

Our cell culture results are further supported by recent phosphorylation analyses using large-scale mass spectrometry approaches showing that Ser-328, Ser-331, and Thr-397, which can be phosphorylated by Chk1 *in vitro,* are also phosphorylated *in vivo* and that phosphorylation of these sites can be observed in response to cell cycle related signaling [91]–[99].

A tight physiological interaction between Chk1 and CK1 isoforms is furthermore indicated by the fact that both kinases are implicated in regulation of DNA damage response and replication by modulating the activity of various regulatory proteins by site-specific phosphorylation in concert. For example, phosphorylation of Cdc25A (cell division cycle 25 A) by both, CK1 (isoforms α and ε) and Chk1, contributes to Cdc25A degradation and precise control of the cell cycle [102], [103]. Furthermore, CK1γ1 has been shown to phosphorylate residues in claspin which are important for Chk1 activation by ATR (ataxia telangiectasia and rad-3-related kinase) [104]. Given these facts, a direct regulatory connection between Chk1 and CK1 in fine tuning of cell cycle regulation is quite plausible.

In summary, we clearly identified phosphorylation sites within the C-terminal domain of CK1δ which can be targeted by Chk1. Furthermore, our results show for the first time that site-specific phosphorylation of CK1δ by Chk1 plays an important role in modulating the activity of CK1δ *in vitro* and most likely also within cellular signal transduction regulation. However, detailed information about this interaction remains to be determined by future work.

## Supporting Information

Figure S1 **Chk1- and CK1δ-specific kinase activity in HT1080 cells is altered after treatment with hydroxyurea.** Cellular Chk1 was activated by treating HT1080 cells with 2.5 mM hydroxyurea (HU) for the indicated periods of time. Immunoprecipitated Chk1 and CK1δ were used to phosphorylate CHKtide substrate peptide (Chk1) or GST-p53^1–64^ (FP267; CK1δ). Substrate phosphorylation was quantified by Cherenkov counting. Data are presented as normalized bar graph.
(TIF)

Figure S2 **CK1δ is inhibited by SB-218078 in vitro.** Kinase activity of rat CK1δ (FP449) was assayed in presence of increasing concentrations of the Chk1-specific inhibitor SB-218078. Phosphorylation intensity of the substrate GST-p53^1–64^ (FP267) was quantified by Cherenkov counting. Dose-response data were processed using GraphPad Prism 5.
(TIF)
