# Supplementary figures and images for: CK1δ Kinase Activity Is Modulated by Chk1-Mediated Phosphorylation
Source: PLoS One. 2013 Jul 4;8(7):e68803. doi: 10.1371/journal.pone.0068803 (PMC3701638; doi:10.1371/journal.pone.0068803)

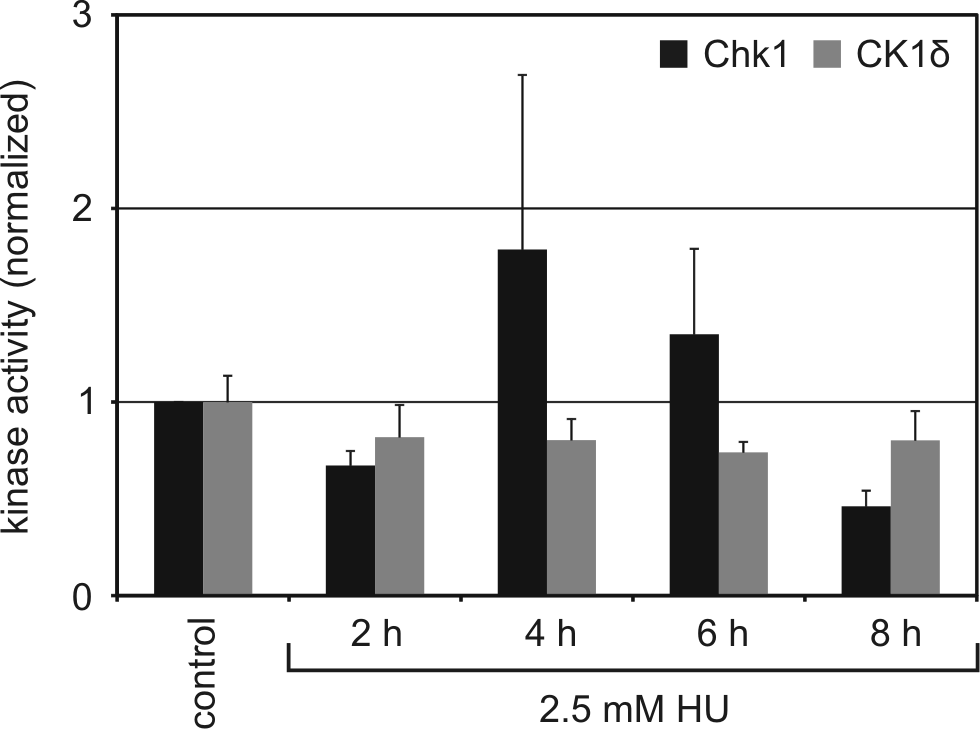

Supplement: Figure S1 — Chk1- and CK1δ-specific kinase activity in HT1080 cells is altered after treatment with hydroxyurea. Cellular Chk1 was activated by treating HT1080 cells with 2.5 mM hydroxyurea (HU) for the indicated periods of time. Immunoprecipitated Chk1 and CK1δ were used to phosphorylate CHKtide substrate peptide (Chk1) or GST-p531–64 (FP267; CK1δ). Substrate phosphorylation was quantified by Cherenkov counting. Data are presented as normalized bar graph. (TIF) [file pone.0068803.s001.tif]

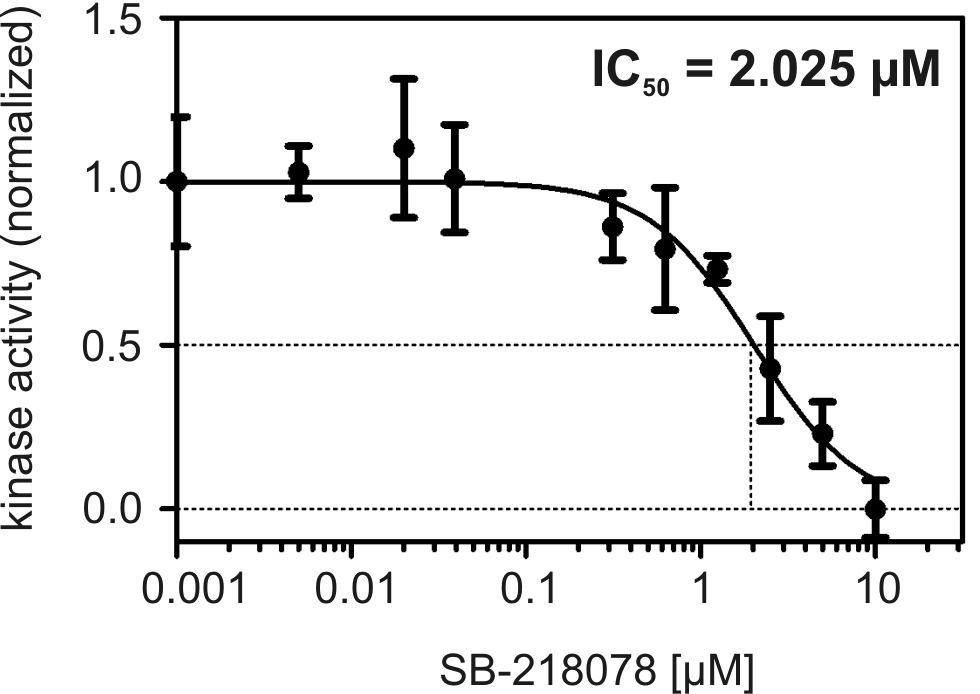

Supplement: Figure S2 — CK1δ is inhibited by SB-218078 in vitro. Kinase activity of rat CK1δ (FP449) was assayed in presence of increasing concentrations of the Chk1-specific inhibitor SB-218078. Phosphorylation intensity of the substrate GST-p531–64 (FP267) was quantified by Cherenkov counting. Dose-response data were processed using GraphPad Prism 5. (TIF) [file pone.0068803.s002.tif]
